# Plasma biomarkers of Alzheimer's disease and cognition in a population‐based sample

**DOI:** 10.1002/alz70861_108402

**Published:** 2025-12-23

**Authors:** Aino Aaltonen, Toni T Saari, Sanna‐Kaisa Herukka, Tarja Kokkola, Sari Kärkkäinen, Karin Lohi, Aarno Palotie, Heiko Runz, Jaakko Kaprio, Valtteri Julkunen, Eero Vuoksimaa, FinnGen

**Affiliations:** ^1^ University of Helsinki, Helsinki Finland; ^2^ University of Eastern Finland, Kuopio Finland

## Abstract

**Background:**

We investigated the associations between several plasma biomarkers of AD and cognition in a population‐based sample of 65–85‐year‐old individuals without prior diagnosis of dementia‐causing neurodegenerative disease.

**Method:**

This cross‐sectional study included 701 twin participants of the TWINGEN study. The data were gathered at six study centers across Finland in 2023. During a research visit, we collected blood samples and participants performed cognitive assessments.

The plasma biomarkers phosphorylated‐tau181 (*p* ‐tau181), phosphorylated‐tau217 (*p* ‐tau217), amyloid beta 1‐42 to amyloid beta 1‐40 ratio (Aβ1‐42/Aβ1‐40), glial fibrillary acidic protein (GFAP) and neurofilament light chain (NfL) levels were measured with Simoa HD‐X Analyzer. A total cognitive score (with higher score indicating better cognition) was based on Episodic Memory Task (learning and recall), and Modified Trail Making A and B Tasks, assessed using web‐based cCOG. We report cross‐biomarker correlations and analyzed associations between biomarkers and cognition with hierarchical linear mixed‐effect models. Covariates included age, sex and education as fixed effects, and family and study site effects as random intercepts. Nested models were compared with likelihood ratio tests, and multiple testing was accounted for using the Benjamini‐Yekutieli correction for False Discovery Rate.

**Result:**

Plasma biomarkers were associated with each other with absolute correlation coefficients ranging from 0.10 between GFAP and Aβ1‐42/Aβ1‐40 to 0.75 between *p* ‐tau181 and *p* ‐tau217. Regarding the associations between biomarkers and cognition, the models including either *p* ‐tau181 or *p* ‐tau217 had statistically significantly better model fit than the model with covariates alone. Adding NfL, GFAP or Aβ1‐42/Aβ1‐40 ratio did not improve model fit over the covariate model. In addition to *p* ‐tau181 (standardized *β* = ‐0.019, 95% CI [‐0.031, ‐0.007]) and *p* ‐tau217 (*β* = ‐0.031, 95% CI [‐0.042, ‐0.020]), NfL had confidence interval differing from 0 (*β* =‐0.014, 95% CI [‐0.025, ‐0.002]).

**Conclusion:**

Plasma *p* ‐tau181 and *p* ‐tau217 were negatively associated with cognition in a population‐based sample of individuals without diagnosis of AD or dementia. Plasma *p* ‐tau has been shown to be associated with longitudinal changes in cognition and to differ between groups of individuals with normal cognition, mild cognitive impairment and AD. Our results highlight the potential value of *p* ‐tau in the detection of early cognitive changes.

